# Spaceflight associated neuro-ocular syndrome: connections with terrestrial eye and brain disorders

**DOI:** 10.3389/fopht.2024.1487992

**Published:** 2024-10-17

**Authors:** Brenna Joe

**Affiliations:** Five Branches University, San Jose, CA, United States

**Keywords:** spaceflight associated neuro-ocular syndrome, traditional Chinese medicine, countermeasure, microgravity, pathophysiology, astronaut

## Abstract

Spaceflight Associated Neuro-ocular Syndrome (SANS) is a series of findings found in astronauts who have experienced long-duration spaceflight. It is characterized by neuro-ocular changes that may irreversibly alter vision and increase the risk for the development of terrestrial eye and brain disorders. Theories regarding its etiology and countermeasures to combat the findings seen continue to evolve. There is currently no direct treatment for SANS. Traditional Chinese Medicine (TCM) modalities have been used to treat eye and brain disorders on Earth that are pathogenically similar to SANS, therefore, TCM may be able to target corresponding pathology in astronauts, prevent and mitigate SANS findings, and decrease the risk for future development of disorders. This paper intends to discuss pathological similarities between SANS and terrestrial eye and brain disorders and how TCM has been used to treat those disorders.

## Introduction

As a result of long-duration spaceflight (LDSF), a unique spectrum of neuro-ocular findings occurs in astronauts, known as Spaceflight Associated Neuro-ocular Syndrome (SANS) (1). The symptoms of SANS can include the following: optic disc edema (ODE), choroidal folds, optic nerve sheath (ONS) distention, hyperopic shift, cotton-wool spots, and posterior globe flattening (GF) (2). Mader et al. estimated that about 23% and 48% of astronauts aboard short-duration and long-duration missions reported visual changes (3). More recently, according to Laurie et al., the incidence of SANS among long-duration astronauts is 70% (4). To this day, the etiology of SANS is not completely understood, however, several hypotheses have been put forward in order to explain its pathogenesis, along with potential countermeasures to mitigate SANS risk. An accumulation of multiple factors is likely contributing to the development and variability of SANS in astronauts that include genetic, anatomic, and spaceflight-related factors (4).

It was initially hypothesized that these findings were the result of an increase in intracranial pressure (ICP) from a cephalad fluid shift due to microgravity (5). The redistribution of fluid is thought to cause venous stasis in the head and neck, leading to inhibited CSF outflow, and thus, elevated ICP (3). A more novel approach has been centered around the glymphatic system, a brain-wide clearance system, where an imbalance can occur around the optic nerve head (ONH), leading to fluid stasis (6). Many countermeasures have been developed and are continuing to evolve for future space application. There are currently no inflight pharmaceutical treatments for SANS that have proven to be effective, although one astronaut received Acetazolamide postflight to decrease cerebral pressure (2, 7). Broadly speaking, when compared with Western medicine, Traditional Chinese Medicine (TCM) can offer alternatives to pharmaceuticals, because acupuncture does not involve the injection of drugs into the body and herbs are often milder and tend to have less side effects, as they can be used in conjunction with medications (8).

In modern times, acupuncture is described as the practice of inserting needles into particular sites on the surface of the body for the purpose of obtaining a therapeutic effect (9). Along with acupuncture, Chinese herbal medicine is a major component of TCM, where the underlying theory of the Chinese materia medica describes many aspects of herbs such as taste, property, channel tropism, and compatibility that are linked with corresponding therapeutic effects (10). Single herbs can also be combined to comprise an herbal formula. Acupuncture has the ability to regulate nerve function at varying levels, including the excitation of peripheral afferent nerve fibers, which may be transferred to the brain through the spinal cord (11). It can also affect the vision area of the brain and elevate intraocular blood flow (12, 13). More specifically, acupuncture may improve neural networks and damage to visual function, along with enhancing the activities of the rod and cone cells of the retina (14). Meanwhile, Chinese herbal medicine can play a key role in neurogenesis, the process of generating functional neurons via neural stem cells (15, 16). A number of Chinese herbs have a neuroprotective effect, decreasing oxidative stress and inflammatory responses (17). TCM has been explored as a means to maintain astronaut health. For example, herbal formulas such as Bu Zhong Yi Qi Tang have been studied, in order to protect astronauts against weightlessness induced muscle atrophy, while Taikong Yangxin has been used to prevent microvascular function changes (18, 19). Since the principles of TCM emphasize the overall balance of the body, and weightlessness induced physiological changes occur in astronauts, which arise due to abnormal changes in season, time, and Earth’s gravity, the application of TCM can be suitable for space medicine (20).

## Glaucoma and SANS

Some of the pathology of SANS not only have similarities with but may increase an astronaut’s risk of developing terrestrial eye and brain conditions (**Table 1**). In glaucoma, the ONH, specifically the lamina cribrosa, is a site of vulnerability, and across this structure, the pressure difference between the intraocular pressure (IOP) and the ICP is the translaminar pressure gradient (TLPG) (21, 22). An imbalance in the TLPG can result in elevated stress on the optic nerve (ON), leading to the development of glaucoma (23). In this region, retinal ganglion cell (RGC) axons leave the globe, entering the ON, and with the strain caused by elevated IOP, damage to the axon can occur, leading to a loss of its function, degenerative atrophy of RGC bodies, and a loss of vision (24, 25).

**Table 1:** Summary of the connections between Spaceflight Associated Neuro-ocular Syndrome (SANS) and terrestrial eye and brain disorders, and Traditional Chinese Medicine (TCM) treatments.

Terrestrial Eye and Brain Disorders	Connection(s) with Spaceflight Associated Neuro-ocular Syndrome (SANS)	Traditional Chinese Medicine (TCM) Treatment(s)
Glaucoma	Increase in intraocular pressure (IOP) (26, 29)	Acupuncture, electroacupuncture, acupressure, transcutaneous electrical nerve stimulation (TENS) (31–33)
Nonarteritic Ischemic Optic Neuropathy (NAION)	Choroidal thickening (38)	Acupuncture (40)
Alzheimer’s Disease	Perivascular spaces (PVS) become enlarged, glymphatic system dysregulation (6, 50, 51)	Electroacupuncture, herbal medicine (52, 53)
Cerebral Edema	Increase in intracranial pressure (ICP) (64, 65) Decrease in cerebral spinal fluid (CSF) resorption (62, 67)	Herbal medicine (68)

Upon entering microgravity, there is an initial rise in IOP, however, after several days, this increase will revert to roughly baseline levels as seen on Earth (26). Despite a persistent upwards displacement of fluid, astronauts involved in extended International Space Station (ISS) missions have not shown a constant increase in IOP, suggesting a compensatory mechanism that normalizes IOP (27, 28). It can be noted that there is currently no published research that has directly commented on the consequences of a temporary rise in IOP (26). Although the use of swimming goggles has been proposed as a mitigation strategy to create an artificial increase in IOP to rebalance the TLPG, since there is a suspected decrease in this gradient due to a larger increase in ICP (28). Swimming goggles are able to raise IOP to a small degree (~3 mmHg), which has the potential to compress the lamina cribrosa and increase the likelihood of damage to the RGC axons, as seen in glaucoma patients (29). IOP has remained elevated in a small quantity of individuals in microgravity compared with preflight values, and reverted to baseline postflight, therefore, those who may be analogous anatomically could be at higher risk during LDSF (26). In this case, a 1G (Earth) environment is necessary to return to baseline levels of IOP, and further investigation is required to determine whether or not IOP levels will revert to baseline in a 0.33G (Mars) or 0.16G (Moon) environment (26). Furthermore, the temporary increase in IOP and the potential development of glaucoma may be influenced by a number of factors upon entering microgravity, namely a cephalad fluid shift that can affect the fluid dynamics and drainage of the aqueous humor, changes in ocular shape and volume (e.g. GF, ODE), and blood flow disturbances to the ON and retina that could result in ischemic damage (28). There are challenges present in treating ocular conditions, which typically require eye drops, and astronauts have reported difficulty in its sterile administration in space (30).

Modalities such as acupuncture, electroacupuncture, acupressure, and transcutaneous electrical nerve stimulation (TENS) have been shown to decrease IOP (31–33). Electroacupuncture involves electrically stimulating needles following insertion, while acupressure may involve massage or stimulator tapping. The mechanism by which IOP decreases could be a result of regulation in the autonomic nervous system (32). The use of acupuncture and electroacupuncture in space is highly unlikely due to limitations and cabin safety. Despite this, acupressure and TENS may be beneficial treatments for astronauts to potentially help prevent the initial elevation of IOP when entering microgravity, decreasing the risk for glaucoma development. During spaceflight, Chinese astronauts have used portable electrical acupoint stimulation devices as a means to apply treatment (34). Variability also exists among astronauts who may have increased susceptibility to developing ocular diseases or have anatomical or physiological disparities within ocular structures, therefore, in the event of glaucomatous damage following spaceflight, these TCM modalities may be helpful in controlling IOP and preventing its progression.

## Nonarteritic anterior ischemic optic neuropathy and SANS

In NAION, circulatory insufficiency is thought to occur within the ONH, and in some cases, general hypoperfusion, or disc or lamina capillary obstruction may occur (35). This leads to ODE and irreparable loss of vision as a result of damage to RGCs (36). Thus, patients will often also experience characteristic visual field defects, a hyperemic optic disc, and peripapillary retinal hemorrhages (35). NAION may be associated with choroidal thickening, which could lead to a compartment syndrome within the ONH, where the axons and their corresponding supply of blood are strained from alterations in choroidal volume (37). Choroidal thickening has also been seen in astronauts early in spaceflight and has persisted the duration of a mission (38). Choroidal expansion could possibly result in ODE (39). The ONH is highly vascularized and the hemodynamics of the microvasculature could be altered with the chronic increase of CSF or tissue fluid leading to swelling of RGC axons (38). Acupuncture has been able to improve the visual function of patients with NAION (40). It is speculated that acupuncture could promote the circulation of blood around the optic disc and arteries in the eyes and brain, enhance the excitability of ON cells that were previously damaged, and restore the visual pathways (40). Acupuncture may be useful to prevent the degeneration of RGCs and repair possible damage incurred to visual pathways in astronauts.

### Alzheimer’s disease and SANS

Alzheimer’s Disease is a neurodegenerative disorder, where its pathological hallmark is an accumulation of beta-amyloid (A β ) and tau proteins (41). A β comes from amyloid precursor protein, created during neuronal activity, and is thought to accumulate due to an imbalance between its production and clearance (42). Findings have indicated there exists a brain-wide clearance system, known as the glymphatic system, that supports interstitial solute, including A β , and fluid clearance (43). The glymphatic system uses polarized aquaporin-4 (AQP4) water channels situated at astrocytic endfeet to clear A β from the brain (43). Damage to the drainage function of the glymphatic system can lead to the failure of clearing toxic proteins, leading to A β buildup (44).

Perivascular spaces (PVS), also known as Virchow-Robin spaces, belong to the glymphatic system, with speculations that the enlargement of PVS may be secondary to A β accumulation and could be an additional marker for neurodegenerative pathology (45). The glymphatic system enables the movement of CSF via a combination of advection and diffusion along periarterial spaces from the subarachnoid space, where it exchanges with interstitial fluid (ISF) within the parenchyma before exiting through perivenous spaces and draining into the peripheral lymphatic system (46). Alterations in the efficiency of glymphatic flow can occur due to reduced AQP4 expression (41). Wang et al. have also provided evidence of an ocular glymphatic system facilitated by AQP4, where A β , produced from retinal neurons, was cleared from the retina and vitreous and directed by the ocular-cranial pressure difference (47). Furthermore, at the ONH, the lamina cribrosa seems to act as a hydrostatic barrier redirecting the movement of fluids and solutes into axons and perivenous spaces (48). Wang et al. additionally used two distinct murine models of glaucoma following normalized IOP and their results indicated excessive and misdirected glymphatic outflow in the glaucomatous eyes, which may ultimately lead to RGC degeneration (47). Therefore, glaucoma may occur without elevated IOP when there is damage to the lamina cribrosa (47). Patients with normal tension glaucoma develop glaucomatous damage in the absence of high IOP, and it is speculated that dysfunction of the ocular glymphatic system, including failure of fluid transport and decreased perivascular waste clearance, may be involved (49).

In space, PVS become enlarged as a result of prolonged exposure to microgravity, altering CSF-ISF circulation and may impair the glymphatic system (50). Wostyn et al. have hypothesized that the enlargement of the PVS in astronauts may be due to altered hemodynamics and decreased CSF outflow, which can give rise to glymphatic perivenous outflow obstruction and increased periarterial CSF inflow, leading to an overflow of CSF along the ONS and elevation of pressure (6). Dysregulation of the glymphatic system may lead to the development of SANS-associated ODE (6). Meanwhile, zu Eulenburg et al. found that the levels of two A β proteins (A β _40_, A β _42_) increased in the blood samples of cosmonauts following LDSF (51). The results further indicated that the A β 42/40 ratio exhibited a downward trend after return to Earth, and generally, this type of trend is not favorable when considering long-term brain health (51). If later confirmed by future studies, dysfunction of the brain glymphatic system of astronauts may lead to increased risk for developing neurodegenerative conditions (6).

Electroacupuncture and herbs have been able to target the glymphatic system (52, 53). Electroacupuncture may decrease the accumulation of A β by improving the clearance function of the glymphatic system (53). Specifically, it has been able to accelerate the paravascular CSF-ISF exchange, impede astrocyte reactivity, and maintain the polarity of AQP4 in the endfeet, improving the drainage function of the glymphatic system and cognitive ability (53). Meanwhile, the herbal formula Yuan Zhi Powder (YZP) could also promote the clearance of the glymphatic system, along with decreasing Aβ deposition (reduced A β _40_, A β _42_ levels), restoring AQP4 polarization, enhancing the drainage function of the meningeal lymphatic vessels, and improving cognitive abnormalities (52). YZP may ultimately function as neuroprotection against pathological damage and cognitive deficits (52). Electroacupuncture may be helpful for astronauts preflight and postflight. When performed preflight, it could potentially promote optimal glymphatic system function, acting as a preventative treatment to decrease the potential severity of A β accumulation. During postflight, electroacupuncture may aid in recovery, enhancing the body’s restoration process and lowering the risk of developing future neurogenerative disorders. Herbal formulas could provide similar effects as electroacupuncture, but can additionally be used during spaceflight.

### Cerebral edema and SANS

Cerebral edema is a pathological accumulation of water in the brain, which exerts pressure within the skull and can lead to elevated ICP (54). The movement of edematous fluids is due to bulk flow, which is directed by the fluid itself, and is primarily driven by hydrostatic and osmotic forces (54). The glymphatic system is hypothesized to play a role in cerebral edema, specifically in cytotoxic edema, in which a reduction in energy enhances the influx of glymphatic CSF and inhibits ISF efflux (55). Meanwhile, interstitial edema can affect patients with hydrocephalus, where CSF accumulates in the cerebral ventricles (56, 57). Idiopathic normal pressure hydrocephalus has been associated with reduced glymphatic system efficiency, but in this case, ventricular enlargement occurs without an elevation of CSF pressure (58–60). Increased lateral and third ventricle volume and elevated aqueductal CSF stroke volume were detected following spaceflight (61, 62). Longer missions have been linked with greater ventricular enlargement, especially throughout the first six months in space, then seemed to taper off (63). Findings have speculated that ventricular expansion might be a compensatory mechanism during spaceflight, in order for the brain to accommodate the cephalad fluid shift, therefore, a period of at least three years may be necessary for ventricular recovery and regaining compensatory capacity (63).

The pathophysiology of cerebral edema has been explored by Galdamez et al. to apply its principles in further understanding the etiology of ODE in SANS (64). Upon entering microgravity, a cephalad fluid shift is thought to cause venous stasis resulting in increased ICP, but findings have supported that ICP may not be pathologically elevated, and only slightly elevated within its normal range (65). Lawley et al. suggested that fluid shifts may seem to inhibit the lowering of ICP, which typically occurs in the terrestrial upright position (66). Nonetheless, a transient increase in ICP that ultimately resolves could instigate a continuous cascade of local inflammation and oxidative stress (64). In addition, decreased CSF reabsorption and lymphatic drainage are mechanisms that could provoke cerebral edema (67). An upward shift in the brain has been seen in astronauts that could result in compression of the superior sagittal sinus, causing impairment of CSF resorption (62).

A Japanese Kampo medicine, called Goreisan (GRS), has been used to mitigate cerebral edema (68). GRS could decrease water influx into the brain, through underlying mechanisms that may include inhibition of AQP4 function, therefore, GRS may improve water maldistribution in the brain, alleviating cerebral edema as well as headaches (68). Spaceflight can provoke headaches in astronauts, most often migraine type during the first week, and tension type in later stages of flight, occurring less frequently (69). With a prolonged stay in space, fluid shifts leading to increased ICP could trigger headaches (69). The action mechanism of GRS on headaches in relation to water maldistribution may involve glymphatic system normalization to clear CSF and inflammatory substances from the brain (68). GRS may be able to promote proper water metabolism and regulate the glymphatic system, preventing stasis and accumulation of water, which may be helpful for astronauts exhibiting SANS findings.

## Discussion and conclusions

**Figure 1:**
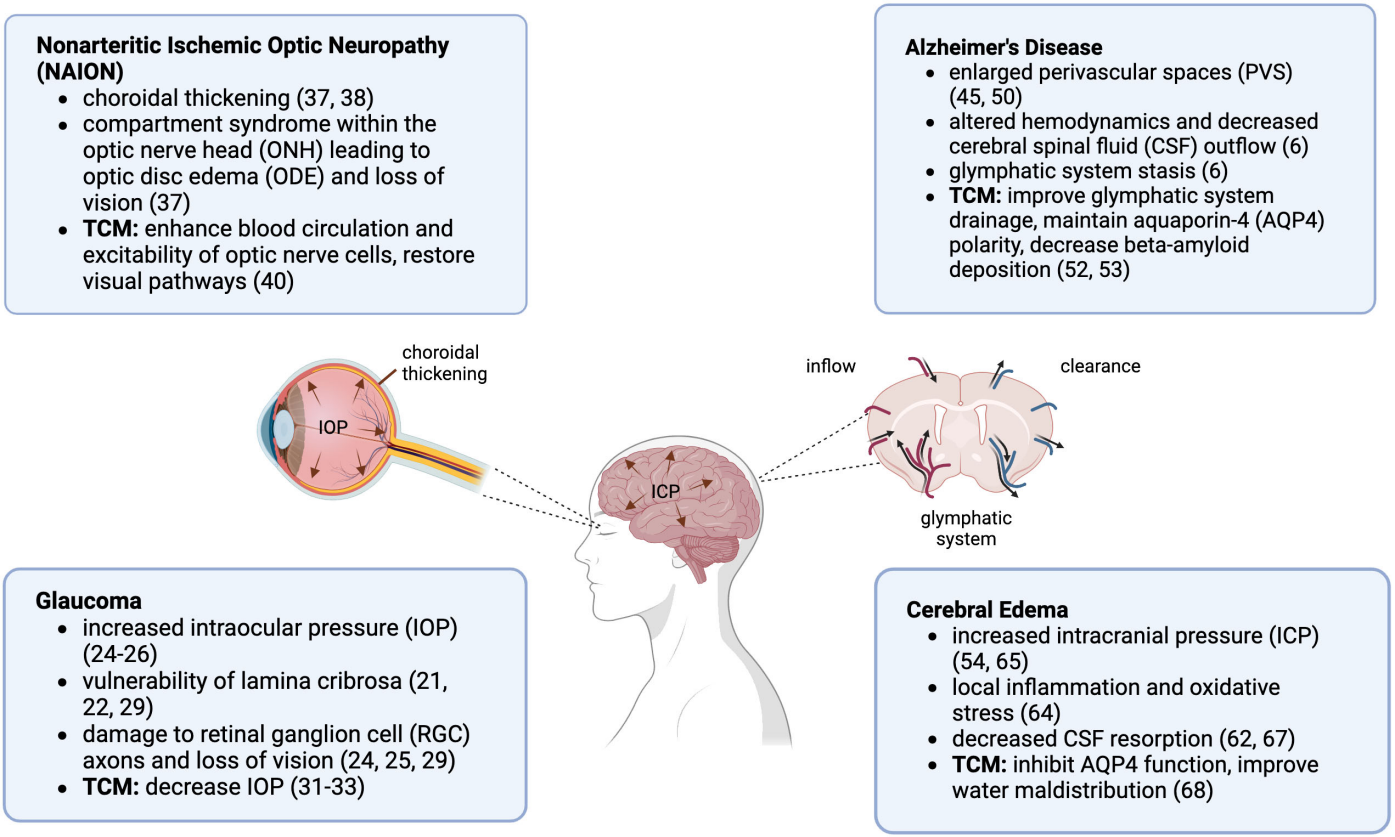
Spaceflight Associated Neuro-ocular Syndrome (SANS) and Traditional Chinese Medicine (TCM) neuro-ocular indications. Created in BioRender.com (71).

Longer duration missions are on the rise and neuro-ocular changes as a result of spaceflight are undeniable. The complete etiology of SANS remains unknown, and theories that have been proposed are not considered mutually exclusive (70). Further understanding of the pathological processes involved in SANS and physiologic risks, and establishing additional countermeasures are imperative to ensure astronaut safety and mission success. Looking at terrestrial eye and brain disorders may provide some insight on the pathogenesis of SANS and give ideas for ways to treat and reduce its risk (**Figure 1**). Studies have demonstrated that astronauts may present with persistent ocular changes well after return from space. Post spaceflight, ODE can occur up to 180 days, while retinal abnormalities can be seen up to 630 days, and GF can persist up to 7 years (72).

Thus far, important progress has been made in the development of countermeasures that continue to evolve for space application. Lower body negative pressure at low levels is one approach, as it has the ability to reverse the cephalad fluid shift, by encircling the legs and pelvic area while providing negative pressure to draw fluid downwards (73, 74). Venous-constrictive thigh cuffs and artificial gravity have also been used to alleviate symptoms related with cephalad fluid shift (75, 76). Other approaches include the use of swimming goggles as a mitigation strategy for SANS to rebalance the TLPG and vitamin B supplements, which may be used to decrease edema and improve microvascular function (4, 29). Meanwhile, incorporating TCM for space application as an alternative approach may not only target pathology, but could prevent and mitigate potential downstream development of SANS in astronauts. In addition, it may be able to reduce the risk for development of eye and brain disorders on Earth after return from spaceflight. TCM is a medicine that not only focuses on the treatment of diseases but emphasizes prevention and constant maintenance to keep the body balanced. It may have great potential to prepare astronauts for LDSF and preserve their long-term health during and post spaceflight with the confirmation from future studies. There are still several unanswered questions regarding SANS. Further research examining the physiologic mechanisms involved in SANS and corresponding terrestrial eye and brain disorders will be helpful to advance physical interventions and preventative treatments. The efforts put forth in expanding knowledge and research in this area can also be beneficial for patients with eye and brain disorders.

## Data Availability

The original contributions presented in the study are included in the article/supplementary material. Further inquiries can be directed to the corresponding author.
